# Establishment and Analysis of Cancer Stem-Like and Non-Cancer Stem-Like Clone Cells from the Human Colon Cancer Cell Line SW480

**DOI:** 10.1371/journal.pone.0158903

**Published:** 2016-07-14

**Authors:** Akari Takaya, Yoshihiko Hirohashi, Aiko Murai, Rena Morita, Hiroshi Saijo, Eri Yamamoto, Terufumi Kubo, Munehide Nakatsugawa, Takayuki Kanaseki, Tomohide Tsukahara, Yasuaki Tamura, Ichiro Takemasa, Toru Kondo, Noriyuki Sato, Toshihiko Torigoe

**Affiliations:** 1 Department of Pathology, Sapporo Medical University School of Medicine, Sapporo, 060–8556, Japan; 2 Department of Respiratory Medicine and Allergology, Sapporo Medical University School of Medicine, Sapporo, 060–8543, Japan; 3 Department of Molecular Therapeutics, Center for Food & Medical Innovation, Hokkaido University, Sapporo, 060–8638, Japan; 4 Department of Surgery, Sapporo Medical University School of Medicine, Sapporo, 060–8543, Japan; 5 Division of Stem Cell Biology, Institute for Genetic Medicine, Hokkaido University, Sapporo, 060–8638, Japan; Cedars-Sinai Medical Center, UNITED STATES

## Abstract

Human cancer stem-like cells (CSCs)/cancer-initiating cells (CICs) can be isolated as side population (SP) cells, aldehyde dehydrogenase high (ALDH^high^) cells or cell surface marker-positive cells including CD44^+^ cells and CD133^+^ cells. CSCs/CICs and non-CSCs/CICs are unstable in *in vitro* culture, and CSCs/CICs can differentiate into non-CSCs/CICs and some non-CSCs/CICs can dedifferentiate into CSCs/CICs. Therefore, experiments using a large amount of CSCs/CICs are technically very difficult. In this study, we isolated single cell clones from SP cells and main population (MP) cells derived from the human colon cancer cell line SW480. SP analysis revealed that SP clone cells had relatively high percentages of SP cells, whereas MP clone cells showed very few SP cells, and the phenotypes were sustainable for more than 2 months of *in vitro* culture. Xenograft transplantation revealed that SP clone cells have higher tumor-initiating ability than that of MP clone cells and SP clone cell showed higher chemo-resistance compared with MP clone cells. These results indicate that SP clone cells derived from SW480 cells are enriched with CSCs/CICs, whereas MP clone cells are pure non-CSCs/CICs. SP clone cells and MP clone cells are a very stable *in vitro* CSC/CIC-enriched and non-CSC/CIC model for further analysis.

## Introduction

Cancer stem-like cells (CSCs)/cancer-initiating cells (CICs) are defined as a small subpopulation of cancer cells that are endowed with high levels of tumor-initiating ability, self-renewal capacity and differentiation ability [1]. CSCs/CICs are resistant to standard therapies including chemotherapy and radiotherapy. These cells are thus thought to be responsible for recurrence and distant metastasis, and their eradication is essential to cure cancer [2]. Human CSCs/CICs were first isolated from acute myeloid leukemia (AML) as CD34^+^CD38^-^ cells [3]. CSCs/CICs have also been isolated from several solid malignancies as side population (SP) cells, aldehyde dehydrogenase high (ALDH^high^) cells, cell surface marker-positive cells including CD44^+^ cells, CD133^+^ cells and sphere-forming cells. SP cells were shown to be enriched with hematopoietic stem cells [4], and subsequent studies revealed that CSCs/CICs could be isolated as cells from several malignancies including glioma [5], hepatocellular carcinoma [6], lung cancer [7, 8], gastrointestinal cancer [9], ovarian cancer [10, 11], thyroid cancer [12], renal cell carcinoma [13] and malignant lymphoma [14]. SP cells are thus a reasonable source for experiments using CSCs/CICs. However, SP cells are unstable and they can differentiate into MP cells very quickly by *in vitro* culture. CSCs/CICs isolated as other cells including ALDH^high^ cells, CD44^+^ cells and CD133^+^ cells can also differentiate. Therefore, experiments using a large amount of very stable CSCs/CICs are technically very difficult, and the establishment of a stable human CSC/CIC line model is needed for further analysis of human CSCs/CICs.

In this study, we isolated SP and MP cells from the SW480 human colon cancer cell line and established SP clone cells and MP clone cells. SP analysis revealed that SP clone cells include SP cells and MP cells, whereas MP clone cells include only MP cells. SP clone cells showed a relatively dormant cell cycle phase and high tumor-initiating ability compared with those of MP clone cells. Thus, SP clone cells established in this study are stable human colon CSCs/CICs.

## Materials and Methods

### Ethics Statement

Mice were maintained and experimented on in accordance with the guidelines after approval by the Committee of Sapporo Medical University (No.10-032). Any animal found unhealthy or sick was promptly euthanized by using isoflurane (DS pharma animal health, Osaka, Japan) and carbon dioxide. The anesthesia and analgesia was performed using isoflurane for experimental procedure. After experiments, all mice were scarified using isoflurane and carbon dioxide.

### Side Population (SP) Assay

Side population (SP) cells were isolated as described previously using Hoechst 33342 dye (Lonza, Basel, Switzerland) with some modifications [4, 15]. Briefly, cells were resuspended at 1 x 10^6^/mL in pre-warmed DMEM supplemented with 5% FBS. Hoechst 33342 dye was added at a final concentration of 2.5 μg/mL in the presence or absence of verapamil (75 μM; Sigma-Aldrich) and the cells were incubated at 37°C for 60 min or 90 min with intermittent shaking. Analyses and sorting were performed with a FACSAria II cell sorter (Becton Dickinson). The Hoechst33342 dye was excited at 357 nm and its fluorescence was analyzed using dual wave lengths (blue, 402–446 nm; red, 650–670 nm).

### Cells and Establishment of SP Clone Cells and MP Clone Cells

The human colon cancer cell line SW480 was purchased from American Type Culture Collection (ATCC, Manassas, VA, USA) and cultured in Dulbecco's modified Eagle's medium (DMEM; Sigma-Aldrich, St. Louis, MO) supplemented with 10% fetal bovine serum (FBS) at 37°C in a humidified 5% CO_2_ atmosphere. SP cells and MP cells isolated from SW480 cells were plated at a single cell per well in a 96-well plate. Sorted single cells were cultured in DMEM supplemented with 10% FBS, and SP clone cells and MP clone cells were obtained after several weeks of culture. To eliminate cell contamination, SW480 cells and all of the SP clone cells and MP clone cells were confirmed by checking the human leukocyte antigen (HLA) by the PCR-SSP method as described previously [16].

### Cell Cycle Assay

SW480 SP clone cells (SP-A, SP-B, SP-H) and MP clone cells (MP-B, MP-D, MP-K) were enzymatically dissociated by incubation in a trypsin-EDTA solution at 37°C, and spheres were mechanically dissociated by pipetting. The cells were fixed with 70% ethanol and resuspended in PBS containing 250 μg/ml RNase A (Sigma-Aldrich) for 30 minutes at 37°C, followed by staining with 50 μg/ml propidium iodide (PI) for 10 minutes at 4°C in the dark. Stained cells were filtered into a conical tube with a 35 μm nylon filter and analyzed with a FACSCalibur (BD Biosciences, San Jose, CA, USA) and Mod-Fit cell cycle analysis program.

### Xenograft Transplantation in NOD/SCID Mice

SW480 SP clone cells (SP-A, SP-B, SP-H) and MP clone cells (MP-B, MP-D, MP-K) were resuspended at concentrations of 1×10^2^, 1×10^3^ and 1×10^4^, respectively, in phosphate buffered saline and Matrigel (BD Biosciences) mixture (1:1), and they were injected subcutaneously into the right and left mid back areas of anesthetized non-obese diabetic/severe combined immunodeficient (NOD/SCID) female mice (Charles River Laboratory Japan, Yokohama, Japan) at the ages of 4–6 weeks. Tumor growth was monitored weekly, and tumor volume was calculated by XY^2^ / 2 (X = long axis, Y = short axis). Cancer stem cell frequency was calculated by using the web program Extreme Limiting Dilution Analysis (ELDA; http://bioinf.wehi.edu.au/software/elda/) software [17].

### Cell Growth Analysis and Chemo Resistance

To compare the cell growth rates, 10^5^ cells were plated in a 6-well plate and cultured in DMEM (Sigma-Aldrich) supplemented with 10% FBS in a 5% CO_2_ incubator for 1, 2 and 3 days, and the number of cells was counted by Countess® (Life Technologies).

To address sensitivities for chemotherapeutic reagents, SW480 cells, SP clone cells and MP clone cells were incubated with Cisplatin (Wako chemicals, Osaka, Japan) or Docetaxel (Wako chemicals) at several concentrations for 72 hrs. The viability of cells were addressed using WST-8 reagent (Dojindo Molecular Technologies, Tokyo, JAPAN).

### Statistical Analysis

Data are presented as means ± SD. Differences in variables were assessed using Student’s t test.

## Results

### Establishment of SP Clones and MP Clones from the Human Colon Cancer Cell Line SW480

**Fig 1:**
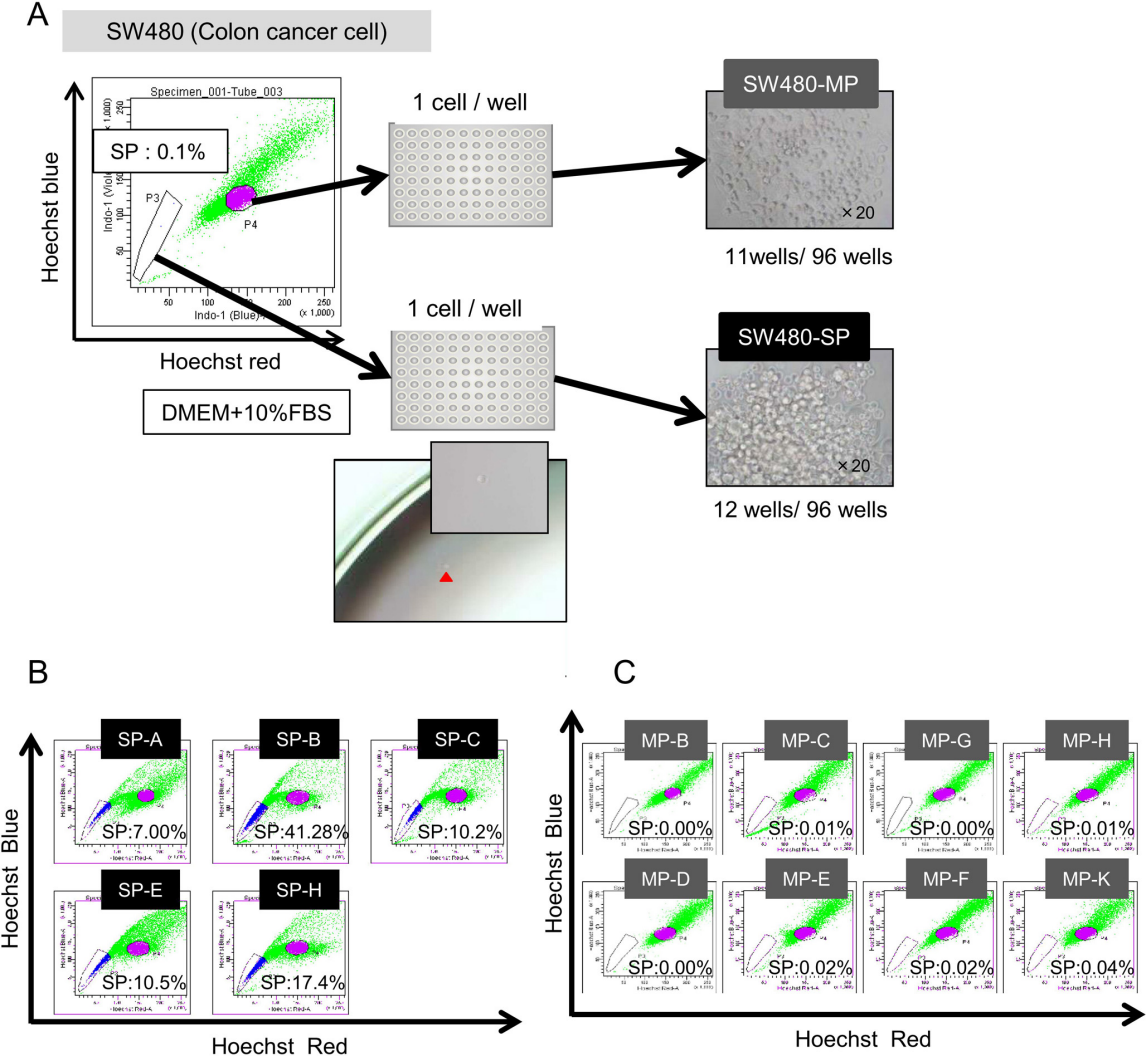
Establishment of SP clones and MP clones from the human colon cancer cell SW480. (A) Summary of establishment of SP clone cells and MP clone cells. SP analysis was performed using SW480 cells. SP cells and MP cells were single cell-sorted using a cell sorter. Single cell platement was confirmed by microscopy. After several weeks of *in vitro* culture, 11 of 96 wells of MP cells and 12 of 96 wells of SP cells showed cell growth. (B) SP analysis of SP clone cells. SP-A, SP-, SP-C, SP-E and SP-H cells were analyzed for SP phenotypes. Percentage indicate the ratio of SP cells. (C) SP analysis of MP clone cells. MP-B, MP-C, MP-D, MP-E, MP-F, MP-G, MP-H and MP-K cells were analyzed for SP phenotypes. Percentage indicate the ratio of SP cells.

Several previous studies showed that CSCs/CICs are enriched in SP cells, not in MP cells, and we previously showed that SP cells derived from SW480 cells are enriched with CSCs/CICs [15]. However, isolation of SP cells takes time and is costly and SP and MP phenotypes are unstable in *in vitro* culture. We therefore aimed to establish stable human CSC/CIC and non-CSC/CIC models in this study. We isolated SP cells and MP cells from SW480 cells and then performed single cell sorting to establish SP clone cells and MP clone cells (Fig 1A). Eleven of 96 wells of MP cell sorting and 12 of 96 wells of SP cell sorting showed cell growth. SP analysis was performed to determine the phenotypes of SP clone cells and MP clone cells. SP clone cells (SP-A, SP-B, SP-C, SP-E, SP-E) showed relatively high percentages of SP cells (7.00% - 41.28%) (Fig 1B), whereas MP clone cells (MP-B, MP-C, MP-D, MP-E, MP-F, MP-G, MP-H, MP-K) showed very low percentages of SP cells (0.00% - 0.04%) (Fig 1C). These results indicate that SP clones contain higher rates of CSCs/CICs, whereas MP clones contain very few CSCs/CICs.

### SP Clones and MP Clones Show Very Stable Phenotypes in *In Vitro* Culture

**Fig 2:**
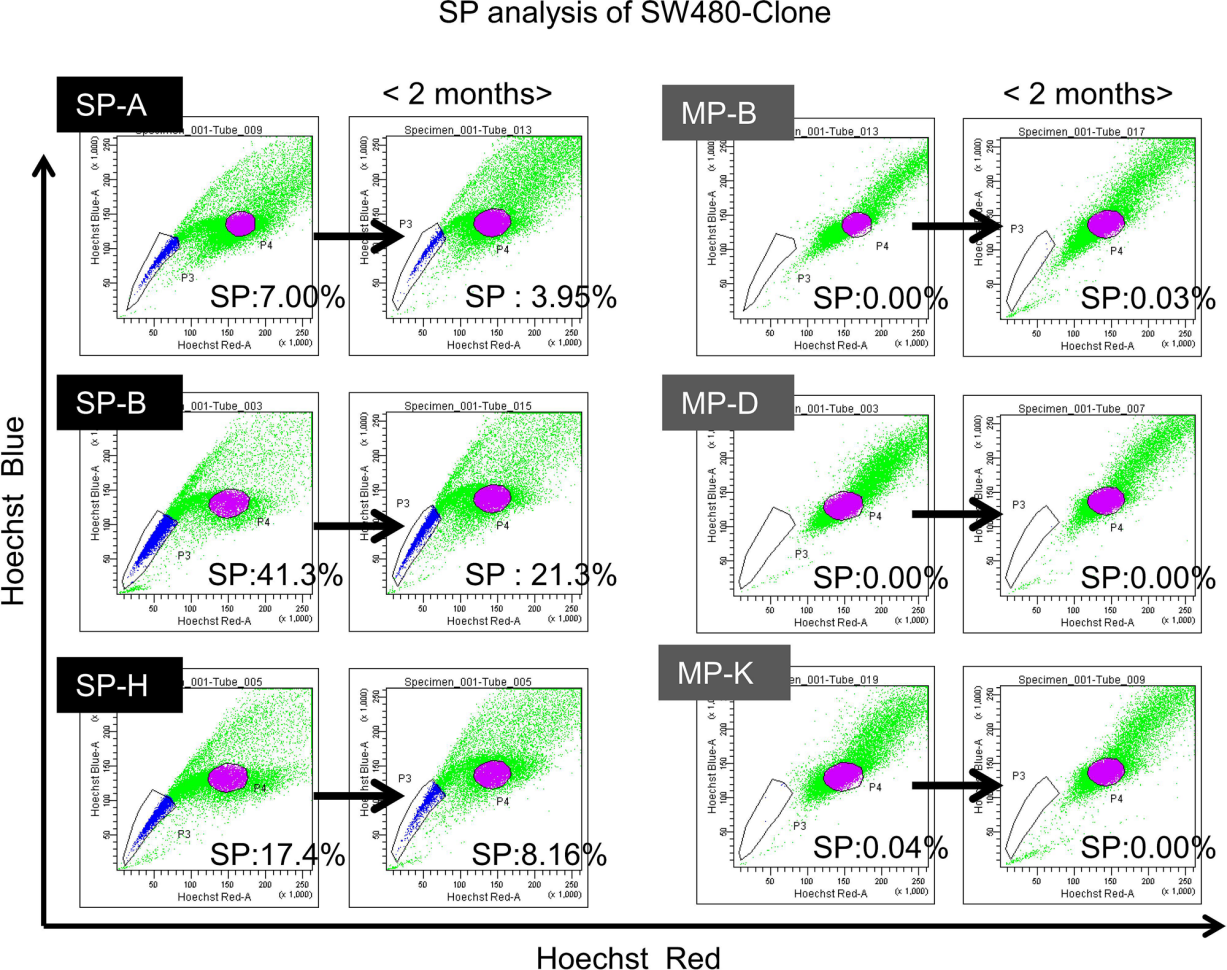
SP clones and MP clones showed very stable phenotypes in *in vitro* culture. SP-A, SP-B, SP-H, MP-B, MP-D and MP-K cells were analyzed for SP phenotypes. The assay was performed before (left) and after (right) 2 months of *in vitro* culture. Percentage indicate the ratio of SP cells.

Since CSCs/CICs can differentiate into non-CSCs/CICs and some non-CSCs/CICs can dedifferentiate into CSCs/CICs by *in vitro* culture, we addressed the stability of SP clone cells and MP clone cells. SP clone cells and MP clone cells were cultured *in vitro* for more than 2 months by a daily cell culture procedure, and SP analysis was performed (Fig 2). Both SP clone cells and MP clone cells sustained the phenotypes for a long *in vitro* culture period, indicating that SP clone cells and MP clone cells are very stable.

### Cancer Stem-Like Cells Are Enriched in SP Clones

**Fig 3:**
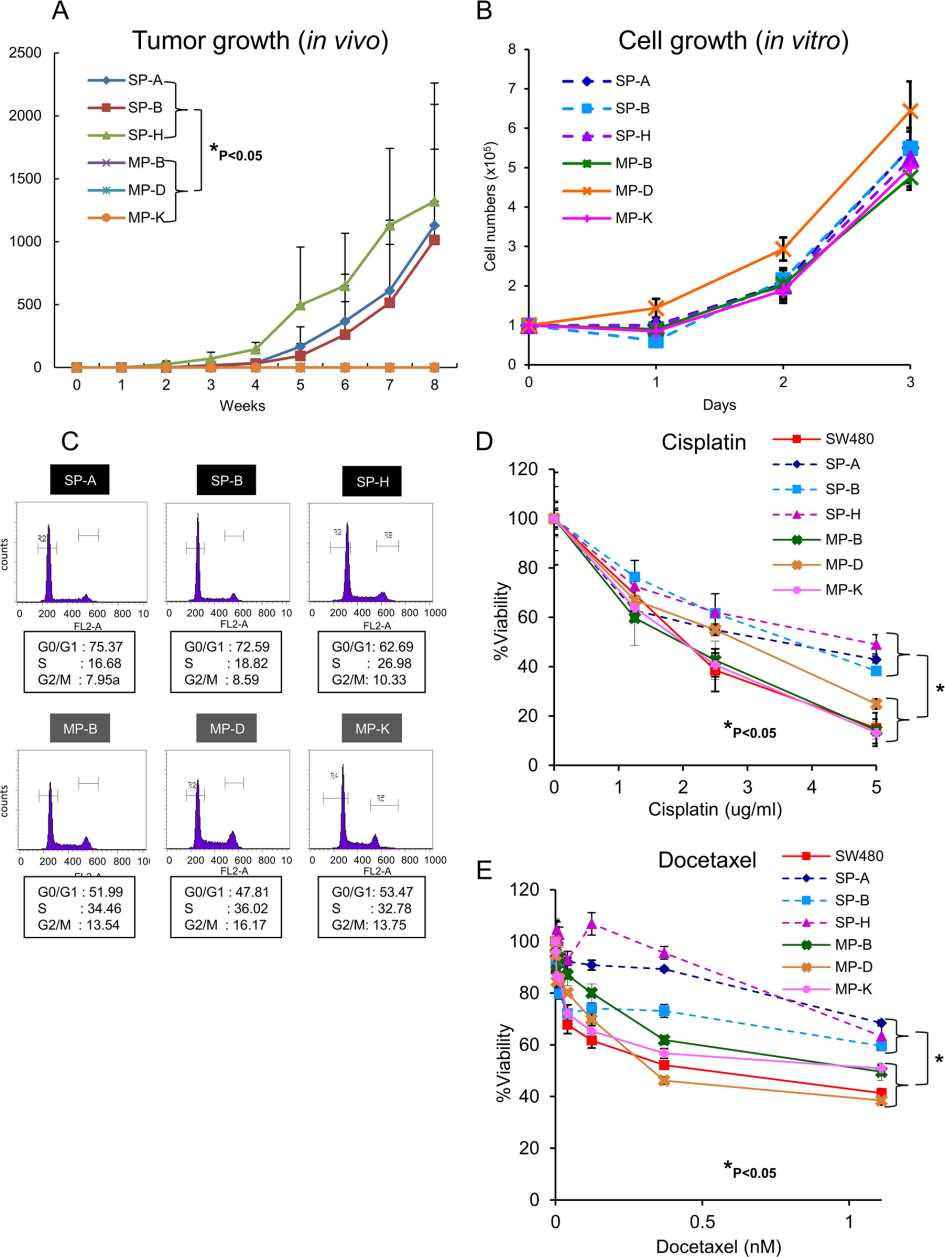
SP clones showed higher percentages of CSCs/CICs than did MP clones. (A) Tumor growth in mice injected with SP clone cells and MP clone cells. SP-A, SP-B, SP-H, MP-B, MP-D and MP-K cells were injected into NOD/SCID mice at 1 x 10^3^ cells/mouse. Data are shown as means ± SD. All statistical analyses for data in this figure were performed using bilateral Student’s t test. P-values: *<0.05. (B) *In vitro* cell growth of SP clone cells and MP clone cells. One thousand of SP clone cells and MP clone cells were seeded and the cell numbers were addressed at day 1, 2, and 3. Data are shown as means ± SD. (C) Cell cycle analysis. The cell cycles of SP-A, SP-B, SP-H, MP-B, MP-D and MP-K cells were. The percentages of cells in G0/G1 phase, S phase and G2/M phase are (D and E) Chemo-resistance. Eight thousand cells were cultured for 3 days in chemotherapeutic reagents (Docetaxel or Cisplatin). The percentage of viability were addressed using WST-8 reagents. Data are shown as means ± SD. Differences between groups were examined for statistical significance by Student's t-test.

CSCs/CICs are defined by their high tumor-initiating ability, and we performed xenograft transplantation using SP clone cells (SP-A SP-B, SP-H) and MP clone cells (MP-B, MP-D, MP-K) using clone cells after more than 2 month *in vitro* culture. SP-H cells showed tumor initiation with transplantation of 1 x 10^2^ cells (1/5), and SP-A and SP-B cells showed tumor initiation with transplantation of 1 x 10^3^ cells (SP-A: 3/5, SP-B: 2/5). On the other hand, only one mouse showed tumor initiation with transplantation of 1 x 10^4^ of MP-B cells, and no tumor initiation was observed with transplantation of MP-B or MP-D cells (Table 1). Tumor growth was significantly faster with SP clone cells compared than with MP clone cells (Fig 3A). The *in vitro* cell growth assay revealed that there are no significant difference between cell growth speed of SP clone cells and MP clone cells (Fig 3B). Cell cycle analysis revealed that percentages of SP clones cells in G0/G1 phase were higher than those of MP clone cells, indicating that SP clone cells are in a relatively dormant state compared with MP clone cells (Fig 3C). SP clone cells showed higher resistance to chemotherapeutic reagents including cisplatin and docetaxel compared with those of MP clone cells (Fig 3D and 3E) that is consistent with previous reports.

**Table 1:** Tumor initiating ability in colon cancer cell line.

		injected cell number				
Cells		10^2^	10^3^	10^4^	CSC/CIC frequency	95%CI
SW480	SP-A	0/5	3/5	3/5	1/5809	2207–15291
	SP-B	0/5	3/5	3/5	1/5809	2207–15291
	SP-H	1/5	2/5	5/5	1/1430	460–4451
	MP-B	0/5	0/5	1/5	1/50335	7134–355168
	MP-D	0/5	0/5	0/5	n.d.	18527-
	MP-K	0/5	0/5	0/5	n.d.	18527-

The analysis was completed 10 weeks following injection. Data are numbers of tumor-initiation/numbers of injections. CSC/CIC frequency and 95% CI were caluculated by ELDA limiting dilution assay. n.d.: not determined. CSC/CIC: cancer stem-like cell/ cancer-initiating cell. 95% CI: Confidence interval.

## Discussion

CSCs/CICs have been isolated by several different methods and analyzed at the molecular level. CSCs/CICs have high tumor-initiating ability and are resistant to standard cancer therapies including chemotherapy, radiotherapy and molecular targeted therapy. Eradication of CSCs/CICs is therefore essential to cure cancer. However, CSCs/CICs also have differentiation ability and they can differentiate into non-CSCs/CICs. Previous studies showed that some types of non-CSCs/CICs can dedifferentiate into CSCs/CICs [18, 19]. Therefore, stable CSC/CIC and non-CSC/CIC models are needed, and we aimed to establish such models in this study. We isolated SP cells from the human colon cancer cell line SW480. In previous studies, we and another group successfully isolated SP cells from SW480 cells that showed a CSC/CIC phenotype [9, 15]. Thus, SP cells derived from SW480 cells are a reasonable source of human colon CSCs/CICs; however, SP cells can differentiate into MP cells in *in vitro* culture and the percentage of SP cells is as low as 0.5%– 3.0%. To establish stable CSC/CIC and non-CSC/CIC line, we performed single cell sorting using 3 difference cancer cell lines including SW480. And we could establish stable SP clone cells and MP clone cells from only SW480 cells. SP clone cells showed relative high percentages of CSCs/CICs as 7.0% - 40.0%, whereas MP clone cells showed pure non-CSCs/CICs. Surprisingly, the phenotypes of SP clone cells and MP clone cells were very stable in *in vitro* culture for more than 2 months. SP clone cells showed higher tumor initiating capability compared with MP clone cells and parental SW480 cells [15], whereas SP clone cells and MP clone cells showed no difference *in vitro* culture speed. Furthermore, SP clone cells showed higher chemo-resistance compared with parental SW480 cells and MP clone cells. These phenotypes highly support that CSCs/CICs are enriched in SP clone cells. Since the phenotypes of SP clone cells and MP clone cells are different, we analyzed cell human leukocyte antigens (HLAs) by the PCR-SSP method to eliminate cell contamination. All SP clones and MP clones showed HLA-A2^+^ and HLA-A24^+^ phenotypes that are compatible with wild-type SW480 cells, indicating that these SP clones and MP clones are derived from SW480 cells. Thus, SP clone cells and MP clone cells are stable CSC/CIC enriched line and stable non-CSC/CIC line, respectively that is ideal material for *in vitro* analysis of CSCs/CICs and non-CSCs/CICs.

A previous study revealed that human colon cancer cells are plastic and that non-CSCs/CICs can undergo dedifferentiation into CSCs/CICs. Activation of the Wnt signal is essential for the plasticity, and contact with myofibroblasts activates the Wnt signal [20]. In this study, MP clone cells showed very low tumor-initiating ability; however, one mouse injected with 1 x 10^4^ of MP-B clone cells showed tumor growth. MP-B clone cells showed a very small percentage of SP cells, indicating that MP-B cells contain almost no CSCs/CICs and that the phenotype is stable. However, cells derived from the tumor of MP-B clone cells were positive for SP cells (data not shown), indicating that some stimulation by the *in vivo* environment induced dedifferentiation of MP-B cells. Activation of Wnt signaling by *in vivo* myofibroblasts may be involved the *in vivo* dedifferentiation. Further analysis of molecular aspects including the Wnt signal of colon cancer plasticity is needed.

In summary, we established SP clone cells and MP clone cells from the human colon cancer cell line SW480. SP clone cells and MP clone cells showed very stable CSC/CIC enriched line and non-CSC/CIC line. This is the first stable human CSC/CIC and non-CSC/CIC model, and this system can be a platform for studies on human CSCs/CICs.
